# Ser/Thr Protein Kinase SpkI Affects Photosynthetic Efficiency in *Synechocystis* sp. PCC 6803 upon Salt Stress

**DOI:** 10.3390/life12050713

**Published:** 2022-05-10

**Authors:** Xiaoting Wang, Haitao Ge, Ye Zhang, Yingchun Wang, Pengpeng Zhang

**Affiliations:** 1 Biotechnology Research Institute, Chinese Academy of Agricultural Sciences, Beijing 100081, China; xiaotingwang2021@163.com (X.W.); zhangyecaas@163.com (Y.Z.); 2 Institute of Genetics and Developmental Biology, Chinese Academy of Sciences, Beijing 100101, China; htge@genetics.ac.cn (H.G.); ycwang@genetics.ac.cn (Y.W.)

**Keywords:** photosystem II, photosynthesis, salt-stress, Ser/Thr protein kinase, *Synechocystis*

## Abstract

High salinity is a common environmental factor that limits productivity and growth for photosynthetic organisms. Here, we identified a mutant defected in gene *sll1770*, which encodes a Ser/Thr protein kinase SpkI, with a significantly low maximal quantum yield of PSII under high salt condition in *Synechocystis* sp. PCC 6803. Physiological characterization demonstrated that the Δ*spkI* mutant had normal growth and photosynthesis under control condition. And a significantly higher NPQ capacity was also observed in Δ*spkI* when grown under control condition. However, when grown under high salt condition, Δ*spkI* exhibited apparently slower growth as well as decreased net photosynthesis and PSII activity. Western blot analysis demonstrated that the amount of major photosynthetic proteins declined sharply in Δ*spkI* when cells grown under high salt condition. Redox kinetics measurement suggested that the activities of PSI and cytochrome *b*_6_*f* complex were modified in Δ*spkI* under high salt condition, which resulted in a more reduced PQ pool in Δ*spkI*. Chlorophyll fluorescence traces suggested that the O_A_^−^ reoxidation and state transition was also impaired in Δ*spkI* under high salt condition. Above all, we propose that Ser/Thr protein kinase SpkI plays a role in maintaining high-effective photosynthesis during high-salt acclimation process in *Synechocystis*.

## 1. Introduction

High salinity is a challenging environmental factor for photosynthetic organisms, such as higher plants, algae and cyanobacteria, as the major reason for decreasing plant productivity by substantially affecting key biological processes and inhibiting photosystem II (PSII) activity as well as causing damages to photosynthetic apparatus in cyanobacteria [1,2]. Thus, maintaining optimal photosynthetic efficiency is an important issue for photosynthetic organisms during high-salt acclimation.

Cyanobacteria are a morphologically and physiologically diverse group of photoautotrophic bacteria that are found in nearly all light-exposed habitats including environments with different salinities such as freshwaters, oceans, and hypersaline ponds or soil surfaces in temperate and arid climates [3]. Cyanobacterial salt acclimation has been investigated in great detail using the model strain *Synechocystis* sp. PCC 6803 (*Synechocystis* 6803). This unicellular cyanobacterium *Synechocystis* 6803 was originally isolated from a freshwater pond [4] but represents a truly euryhaline organism able to grow in freshwater but also in media containing salt concentrations twice as high as in seawater [5]. Previous studies revealed that *Synechocystis* 6803 is able to acclimate in high concentrations of NaCl up to 1.2 M by synthesis of the compatible solutes [6], efflux of inorganic cations via transporters [7], and regulation of main bioenergetic processes, such as photosynthesis and respiration [8]. Expression analyses showed similar response patterns of some salt-induced genes in *Synechocystis* and higher plants. Combined with various friendly features for genetic manipulation, *Synechocystis* is an ideal model for studying salt stress response mechanisms in photosynthetic organisms [9].

Eukaryotic-type Ser/Thr protein kinases were proven to exist widely in prokaryotes, and regulate various cellular functions, such as development [10], stress response [11]. There are a total of 12 genes encoding putative Ser/Thr protein kinases in *Synechocystis*, which can be divided into two subgroups according to their protein features, seven of them belonging to the PKN2 subfamily (spkA-spkG), and the rest five belonging to the ABC1 subfamily (spkH-spkL) [12]. Except SpkE, the rest Ser/Thr kinases were proven with kinase activity, and few substrates have been identified [13]. And some of these Ser/Thr kinases have been reported playing different roles in *Synechocystis* sp. PCC 6803. SpkA and SpkB are essential for cell motility [14], and SpkB also participates in oxidative stress response [15]. SpkC might take part in the regulation of nitrogen metabolism [16]. SpkD might be involved in adjusting the pool of tricarboxylic acid (TCA) cycle metabolites [17]. SpkE may act as an additional component in the regulatory pathway of *Synechocystis* sp. PCC 6803 cells in response to cold stress [18]. SpkG plays an essential role in high-salt resistance [19], and is involved in the phosphorylation of Fd5 (ferredoxin 5) protein in *Synechocystis* [20].

Although applications of modern “omics” technologies have revealed many salt-regulated genes, the signaling and regulation of salt stress response in cyanobacteria is far from well understood. Even though previously reported that most of single kinase mutants didn’t show any obvious phenotypes when grown under standard conditions [21], it’s evident that the significance of Ser/Thr kinases under various stress conditions cannot be neglected. To this end, screening of *Synechocystis* transposon-insertion library was performed using chlorophyll fluorescence technique, and a mutant defect in gene *sll1770* encoding a Ser/Thr protein kinase SpkI with significantly lower Fv/Fm under high salt condition was identified. The physiological importance and possible mechanisms of SpkI involved during high salt acclimation in *Synechocystis* were studied.

## 2. Materials and Methods

### 2.1. Strains and Culture Conditions

The *Synechocystis* sp. PCC 6803 glucose-tolerant strain was used as wild type (WT) in this study. The *spkI* inactivation mutant Δ*spkI* was created by insertion of a kanamycin-resistant cassette at the position 249 of *sll1770*. Construction of the complementary strain P*petJ*::*spkI* was based on the procedure described in Eisenhut et al. [22]. Eventually, a DNA fragment containing the coding region of *spkI* gene behind a *PetJ* promoter was integrated into the *spkA* site of the Δ*spkI* genome. The complete segregation for above strains was verified by PCR, and primers used in this study were listed in Table S1.

The WT, Δ*spkI* and P*petJ*::*spkI* strains were grown photoautotrophically at 30 °C under continuous white light illumination with photon flux density of 50 μmol photons m^−2^ s^−1^. The cells were inoculated either on 1.5% (*w*/*v*) agar plates or in liquid cultures aerated by gently shaking at 120 rpm. The growth medium in control condition was BG-11 buffered with 20 mM HEPES-NaOH (pH 7.5). With regard to high salt treatment, 0.9 M NaCl in liquid and 0.6 M NaCl in solid medium were supplemented.

### 2.2. Transposon-Insertion Mutant Library Construction and Screening

The *Synechocystis* transposon-insertion library was constructed according to Ozaki et al. [23], and then followed by transformation of *Synechocystis* WT cells. The transformants with chloramphenicol resistance and WT cells were plated parallel on solid medium with and without additional 0.6 M NaCl. The chlorophyll fluorescence induction was monitored using FluorCam 800MF (Photon Systems Instruments, Drasov, Czech Republic). The colonies with distinct Fv/Fm on high salt plate but normal on control plate were picked up. The transposon insertion site of each selected mutant was identified by inverse PCR method with primers (Table S1) as described earlier [24].

### 2.3. Oxygen Evolution/Uptake Measurements

Steady-state oxygen evolution and uptake rates were measured with a Clark-type oxygen electrode (Hansatech) at saturating white light intensity (2500 μmol photons m^−2^ s^−1^). The WT, Δ*spkI* and P*petJ*::*spkI* were grown in BG-11 liquid medium (control) and in additional NaCl respectively. The cell suspensions were adjusted to chlorophyll concentration at 5 μg/mL. Net photosynthesis was measured in the presence of 10 mM NaHCO_3_. PSII activity was measured in the presence of 0.5 mM 2,6-dichloro-*p*-benzoquinone (DCBQ) and 1 mM K_3_Fe(CN)_6_. PSI activity was measured in the presence of 20 μM 3-(3,4-dichlorophenyl)-1,1-dimethylurea (DCMU), 1.5 mM methyl viologen, 1 mM sodium ascorbate and 1 mM diaminodurene. Respiration rate was recorded in darkness after illumination for 1 min.

### 2.4. RNA Isolation and Quantification

Total RNA was isolated by Trizol method and treated with DNase (Turbo DNA-free kit, Invitrogen, Carlsbad, CA, USA) to remove genomic DNA. The first-strand cDNA was synthesized from 2.5 μg total RNA using a Revert aid First Strand cDNA Synthesis Kit (Thermo Scientific, Carlsbad, CA, USA). Quantitative Real-Time PCR was performed on an ABI 7500 system. The primers (Table S1) were designed to generate 150–200 bp amplicons. The *rnpB* was used as a reference gene.

### 2.5. Protein Isolation and Immunodetection

The total cell extract of *Synechocystis* was isolated at 4 °C according to Zhang et al. [25]. Cells were harvested and washed twice with a buffer (50 mM HEPES-NaOH pH 7.5, 30 mM CaCl_2_) and then resuspended in a buffer (50 mM HEPES-NaOH pH 7.5, 30 mM CaCl_2_, 800 mM sorbitol and 1 mM 6-aminohexanoic acid). The cells were broken by vortexing 8 × 1 min in the presence of glass beads. The total cell extract was obtained by centrifugation at 2000× *g* twice for 5 min to remove the glass beads and cell debris.

Protein samples were separated and immunodetected according to Zhang et al. [26]. Solubilized proteins were separated by 12% or 15% SDS-PAGE with 6 M urea. After electrophoresis, the proteins were electrotransferred to a polyvinylidene fluoride (PVDF) membrane (Immobilon-P, Millipore, Burlington, MA, USA) by a semidry apparatus and immunodetected with specific antibodies. NdhK antibody was kindly gifted by Weimin Ma from Shanghai Normal University. The D1 (AS10704), PsbD (AS06146), PsbI (AS10692), PsaB (AS10695), PetC (AS08330), APC (AS08277), RbcL (AS03037) and AtpB (AS05085) antibodies were purchased from Agrisera. The PsbB (PHY0319) antibody was purchased from PhytoAB.

### 2.6. Chlorophyll Fluorescence Measurements

Non-photochemical quenching (NPQ) is one of the most important photoprotection mechanisms in cyanobacteria. NPQ measurements were performed using FluorCam 800MF (Photon Systems Instruments, Drasov, Czech Republic). The cells were suspended in the same medium as they grown, and were adjusted to 5 μg/mL chlorophyll. Cells were dark-adapted before subjecting to a blue light (1000 μmol photons m^−2^ s^−1^) for 5 min.

Fluorescence induction and flash-induced fluorescence increase and the subsequent decay of chlorophyll fluorescence yield were performed on a fluorometer FL 3500 (Photon Systems Instruments, Drasov, Czech Republic) according to Zhang et al. [27]. Cell suspensions were dark-adapted for 15 min before measuring. The chlorophyll concentration for fluorescence induction and decay were adjusted to 3 μg/mL and 5 μg/mL respectively. To estimate the oxidoreduction level of PQ pool, the fluorescence was induced by illuminating cells with 100 μmol photons m^−2^ s^−1^ actinic light [28]. Fluorescence decay was monitored after a single saturating flash. For deconvolution of the measured curves, a fitting function with three components F(t) − F_0_ = A1 × exp(−t/T1) + A2 × exp(−t/T2) + A3/(1 + t/T3) was applied.

A Joliot-Type Spectrophotometer (JTS-10, Bio-Logic Science Instruments, Seyssine, France) was used to record chlorophyll fluorescence traces. The cells were adjusted to chlorophyll concentration of 5 μg/mL, and were dark adapted for 10 min before initiation of the experiments. To measure the maximal PSII quantum yield, two different actinic light intensities (630 nm, 45 and 320 μmol photons m^−2^ s^−1^) were applied. The fluorescence parameters were determined as follows: F_v_/F_m_ = (F_m_ − F_0_)/F_m_; F_v_’/F_m_’ = (F_m_’ − F_S_)/F_m_’.

For state transition measurements, dark-adapted cells with chlorophyll concentration of 3 μg/mL were illuminated in turn with red light (45 μmol photons m^−2^ s^−1^), blue light (75 μmol photons m^−2^ s^−1^), and red light (45 μmol photons m^−2^ s^−1^) [28].

### 2.7. P700 and Cytochrome b_6_f Measurements

The oxidation and reduction of PSI and cytochrome *b*_6_*f* complex were measured by JTS-10 (Bio-Logic Science Instruments, Seyssinet, France) with the absorbance mode. The cells were dark-adapted for 15 min and excited with far-red actinic light (725 nm, 1400 μmol photons m^−2^ s^−1^) followed by darkness. For P700 measurements (chlorophyll concentration 10 μg/mL), the absorbance changes at 810 nm were recorded. For the cytochrome *b*_6_*f* activity measurements (chlorophyll concentration 25 μg/mL), the absorbance changes were collected at 546, 554, 563 and 573 nm. The signals of cytochrome *f* and plastocyanin were calculated via built-in software provided by Bio-Logic Science Instruments.

## 3. Results

### 3.1. Identification of SpkI under High Salt Condition

**Figure 1:**
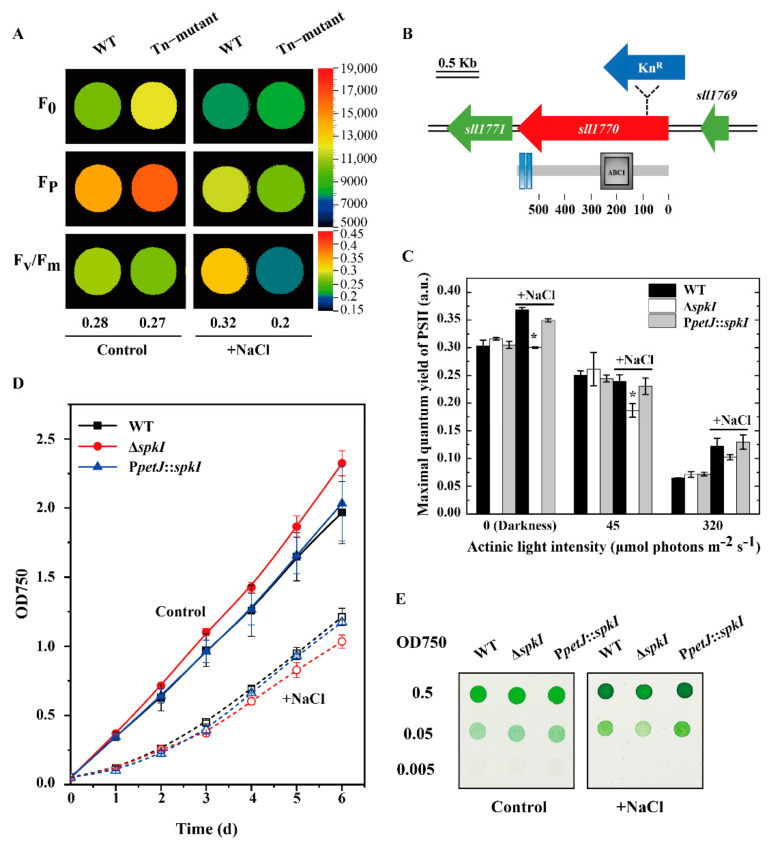
Identification of Δ*spkI* and growth of WT, Δ*spkI* and P*petJ*::*spkI*. (**A**), Chlorophyll fluorescence parameters of WT and Tn–mutant measured by FluorCam. Cell suspensions were adjusted to 5 μg/mL Chl. *a* for all strains before measuring. (**B**), Organization of gene *spkI* (*sll1770*) and construction of Δ*spkI* mutant. Protein features of SpkI, containing an ABC1 domain (position 142–261) and two transmembrane regions (position 530–549 and position 553–575 respectively) were marked below; (**C**), Maximal quantum yields of PSII of WT, Δ*spkI* and P*petJ*::*spkI* in darkness and under illumination; the results were plotted as mean ± S.D. from three independent experiments. Student’s *t* test was applied to analyze significant levels. Asterisk marks significant differences for the mutant Δ*spkI* vs. WT at the level of *p* < 0.05 (*). (**D**), The growth curve of WT, Δ*spkI* and P*petJ*::*spkI* in liquid BG-11 medium monitored by optical intensity at 750 nm; Solid line represents the growth rate measured under control condition, dashed line represents the growth under high salt condition; Open symbols represent the same as the solid symbols; The error bar represents standard deviation (S.D.) from at least 3 independent experiments; the initial OD750 was 0.05. (**E**), The growth of WT, Δ*spkI* and P*petJ*::*spkI* on agar plates. The cells were diluted to 0.5, 0.05, 0.005 at OD750 and quantitatively spotted onto the media, and were photographed after 3 days and 6 days respectively. NaCl concentrations of 0.9 M for liquid cultures and 0.6 M for solid plates were applied to all experiments in our study.

To identify novel genes involved in salt-adaptation mechanisms, a *Synechocystis* mutant displaying a significant lower Fv/Fm value under high salt condition (Figure 1A) was selected by screening a transposon mutant library. The transposon insertion site, located at position 249 of gene *sll1770* was verified by PCR. The gene *sll1770* encodes an ABC1-type Ser/Thr protein kinase SpkI, containing an ABC1 domain and two transmembrane regions. To verify the phenotype, an insertion mutant Δ*spkI* (Figure 1B and Figure S1) and a complementary strain P*petJ*::*spkI* (Figure S2) were constructed and analyzed. Chlorophyll fluorescence parameters showed that the Δ*spkI* had significantly lower values of maximum quantum yield of PSII than those in WT and P*petJ*::*spkI* under high salt condition, both in darkness (Fv/Fm) and in light (Fv’/Fm’) (Figure 1C), which was consistent with the preliminary screening results. Since the phenotype of Δ*spkI* was similar to the transposon mutant (Tn–mutant), we took Δ*spkI* and the complementary strain P*petJ*::*spkI* for further characterization. The cell growth under both conditions was shown in Figure 1D. WT and P*petJ*::*spkI* grew similarly under both conditions, whereas Δ*spkI* grew faster in the control but slower under high salt condition. Similar results were obtained when the cells grew on agar plates (Figure 1E).

The slower growth and lower Fv/Fm of Δ*spkI* under high salt condition suggested defects in photosynthesis, most likely related to PSII activity. Therefore, the activities of net photosynthesis, PSII and PSI as well as respiration of WT, Δ*spkI* and P*petJ*::*spkI* grown under both conditions were measured. When the cells grew under control condition, there were no significant differences on all measured parameters among strains, except noticeably higher PSII activity and lower PSI activity in Δ*spkI*. In additional NaCl, the net photosynthesis rate of all three strains decreased, especially for Δ*spkI* (from 410 to 259), while the PSII activity remained similar to that in the cells grown under control condition except Δ*spkI* (from 770 drop to 530). Compared to WT and complementary strain, Δ*spkI* showed declines of 20% and 30% in the maximal net photosynthesis rate and PSII activity respectively under high salt condition. Additional salt enhanced respiration for all strains, which was more prominent in Δ*spkI*. On the contrary, the PSI activity was not sensitive to high salt condition (Table 1).

### 3.2. Accumulation of Major Photosynthetic Proteins in WT, ΔspkI and PpetJ::spkI

**Figure 2:**
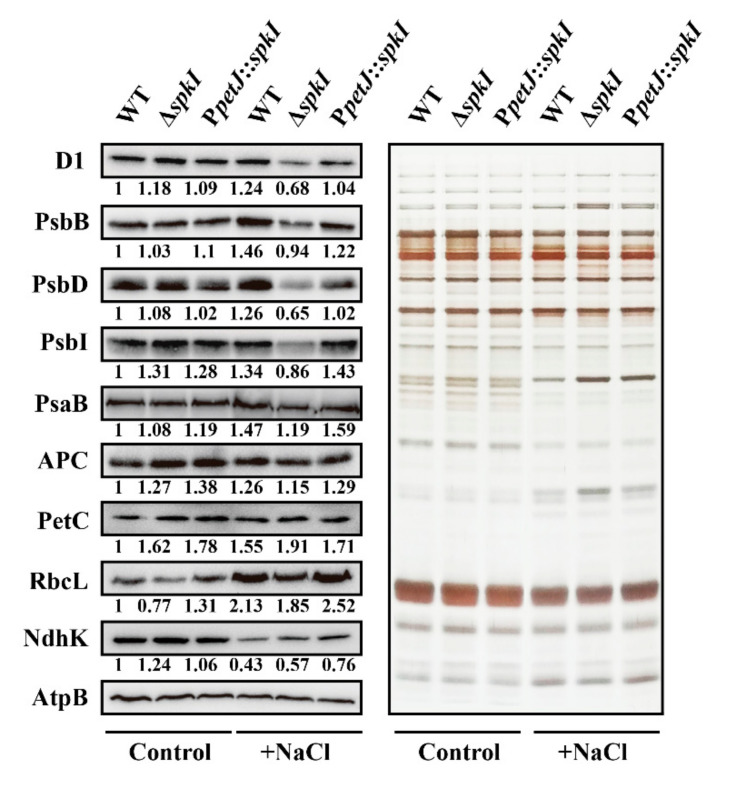
Major photosynthetic proteins in WT, Δ*spkI* and P*petJ*::*spkI*. **Left panel**, Western blots. Total proteins (10 μg in each well) were separated by SDS-PAGE and immuno-detection was performed using D1, PsbB, PsbD, PsbI, PsaB, APC, PetC, RbcL and NdhK specific antibodies, and AtpB was set as an internal reference for membrane proteins. **Right panel**, Silver-stained gel. Total proteins (1 μg in each well) were loaded for SDS-PAGE. The integrated density of protein bands was analyzed by ImageJ tool and normalized with AtpB. The integrated density of each protein in WT under control condition was set as 1.

Since interruption of gene *spkI* altered photosynthesis activity, content of major photosynthetic proteins from total cell extracts was analyzed (Figure 2). In control condition, D1 protein content in Δ*spkI* was slightly higher than that in WT and P*petJ*::*spkI*, but remarkably decreased in high salt condition. Other PSII subunits including PsbB (CP47), PsbD (D2) and PsbI, showed a similar trend. This was in agreement with the oxygen evolution data (Table 1). PSI subunit PsaB and phycobilisome core protein APC were decreased in Δ*spkI* under high salt conditions. Amount of Rieske protein of cytochrome *b*_6_*f* complex PetC was higher in Δ*spkI* than in WT under both conditions. The large subunit of Rubisco RbcL was decreased in Δ*spkI* under both conditions. Increase of RbcL in high salt-exposed cells was probably due to down-regulation of phycobilisome consisting majority of soluble proteins in cyanobacteria under salt stress. NdhK, representing NDH-1 complexes, was down-regulated under high salt condition in all strains. The amount of NdhK was higher in salt-exposed Δ*spkI* than that in WT, suggesting a faster NDH-dependent CET in Δ*spkI*.

### 3.3. Chlorophyll Fluorescence in WT, ΔspkI and PpetJ::spkI

**Figure 3:**
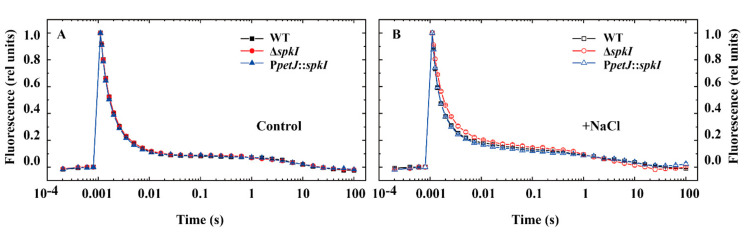
Relaxation of flash-induced chlorophyll fluorescence of WT, Δ*spkI* and P*petJ*::*spkI*. Cell suspensions for all strains were adjusted to 5 μg/mL Chl. *a* and were in darkness for 15 min before measuring. (**A**), Fluorescence decay of strains cultivated under control condition. (**B**), Fluorescence decay of strains cultivated under high salt condition. The error bar represents standard deviation (S.D.) from at least 3 independent experiments.

To investigate changes in PSII performance, the electron transfer of PSII was monitored by kinetics of flash-induced fluorescence decay (Figure 3). The relaxation of the flashed-induced increase in variable chlorophyll fluorescence yield reflects the oxidation of Q_A_^−^ via forward electron transport to Q_B_ (and Q_B_^−^) and charge recombination with donor-side components [29]. The fluorescence decay curves were shown in Figure 3 and the fitting parameters were summarized in Table 2. Under control condition, the curves of three strains were almost identical (Figure 3A), and only minor difference between kinetic parameters was observed (Table 2). Under high salt condition, Δ*spkI* showed a slower kinetics compared with WT and P*petJ*::*spkI* (Figure 3B) with a bigger time constant T1 and a smaller amplitude of fast phase A1 as well as bigger amplitude of middle phase A2 (Table 2). No obvious differences were observed in the slow phase.

**Figure 4:**
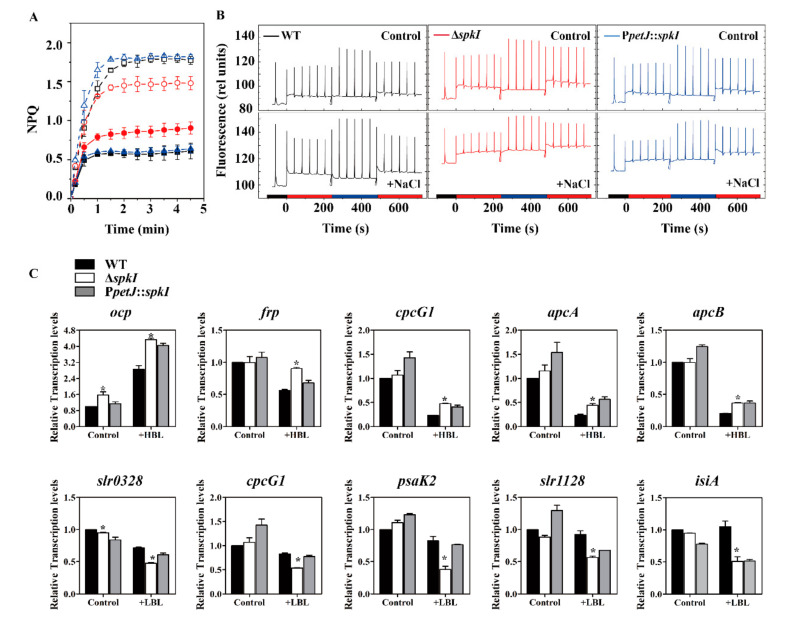
NPQ development and state transition analysis. (**A**), Induction of NPQ of WT, Δ*spkI* and P*petJ*::*spkI* under blue light. Plotted are given as means with error bars. Solid and dash lines and symbols represent cells grown in the control and additional NaCl conditions respectively; Black square symbol: WT, red circle symbol: Δ*spkI*, blue triangle symbol: P*petJ*::*spkI*; (**B**), State transitions of WT, Δ*spkI* and P*petJ*::*spkI* cells grown under both conditions; (**C**), Transcript accumulation of selected genes involved in NPQ and state transitions. The **upper panel** displayed the analysis of genes related to NPQ induction in *Synechocystis*; +HBL: shift of cells from growth light (white, 50 μmol photons m^−2^ s^−1^) to High Blue Light (1000 μmol photons m^−2^ s^−1^) for 5 min; *ocp* (*slr1963*) encodes Orange Carotenoid Protein; *frp* (*slr1964*) encodes Fluorescence Recovery Protein; *cpcG1* (*slr2051*) encodes phycobilisome rod-core linker polypeptide; *apcA* (*slr2067*) encodes allophycocyanin α subunit; *apcB* (*slr1986*) encodes allophycocyanin β subunit. The **lower panel** displayed the analysis of genes involved in state transitions in *Synechocystis*; +LBL: shift of cells from growth light (white, 50 μmol photons m^−2^ s^−1^) to Low Blue Light (80 μmol photons m^−2^ s^−1^) for 5 min; *slr0328* encodes a protein named SynPTP, a low molecular weight protein tyrosine phosphatase; *cpcG1* (*slr2051*) encodes phycobilisome rod-core linker polypeptide; *psaK2* (*sll0629*) encodes alternative photosystem I reaction center subunit X; *slr1128* encodes one component of the high light-inducible carotenoid-binding protein complex; *isiA* (*sll0247*) encodes iron-stress induced chlorophyll-binding protein, also named as CP43′. The abundances of the transcripts were calculated relative to the reference gene *rnpB*. The transcripts of WT in the control were set as 1. Experiments above were repeated for at least three times. Significant difference levels of transcripts in Δ*spkI* vs. WT was analyzed by performing student’s *t* test, with a significance level of *p* < 0.05 (*).

Since PSII of Δ*spkI* was modified especially under salt stress, two main photoprotection mechanisms, NPQ and state transitions, were measured. In the control, the quenching level was much higher in Δ*spkI* than in WT and P*petJ*::*spkI*. Additional NaCl enhanced NPQ level in all strains but the increasement occurred in Δ*spkI* was much less resulting a lower NPQ level in Δ*spkI* under high salt condition (Figure 4A). The state transitions of WT, Δ*spkI* and P*petJ*::*spkI* were monitored in cells grown under both conditions. Δ*spkI* showed a higher F_0_ level under both conditions, and additional NaCl enhanced F_0_ and F_s_ levels overall, especially for Δ*spkI*. Upon light shift of red-blue-red, state transitions were taken placed as State 2-State 1-State 2 in all strains. However, the transition from State 1 to State 2 in Δ*spkI* was not as complete as that in WT. The situation of P*petJ*::*spkI* was similar to WT under control condition and was between WT and Δ*spkI* under high salt condition (Figure 4B).

Since disruption of gene *spkI* led to induction of a higher level of NPQ, transcripts of genes related to NPQ induction in cyanobacteria were analyzed (Figure 4C). The transcripts of most genes accumulated in WT and Δ*spkI* cells grown in control condition were similar, except that of *ocp* was 56% higher in Δ*spkI*, suggesting a higher NPQ capacity in the mutant. Upon shift to strong blue light, transcripts of all selected genes displayed significantly higher accumulations in Δ*spkI* than those in WT. The transcripts of genes encoding phycobilisome (*apcA*, *apcB* and *cpcG1*) declined after strong blue light irradiation, which is to decrease excitation energy reaching to PSII reaction centers. Induction of *ocp* and suppression of *frp* transcripts under blue light could ensure the maximal quenching activity. In addition, shift of standard white-light acclimated cells (State 2) to low blue light will not induce significant NPQ but allow state transitions to take place. The transcriptional levels of five genes involved in state transitions in *Synechocystis* were similar between WT and Δ*spkI* in the control, but were remarkably decreased in Δ*spkI* (43–62%) upon shift to low blue light (Figure 4C), which suggested a probable defect in state transitions in Δ*spkI* when the cells were in State 1 (Figure 4B).

### 3.4. Activities of PSI and Cytochrome b_6_f in WT, ΔspkI and PpetJ::spkI

As modifications of photosynthetic electron transport in Δ*spkI* was proven in PSII under high salt condition (Figure 3), downstream electron transfer of PSI and cytochrome *b*_6_*f* complex in WT, Δ*spkI* and P*petJ*::*spkI* grown under both conditions was investigated. The rates of oxidation and reduction for P700 as well as cytochrome *f* and plastocyanin presented as t_1/2_ were summarized in Table 3.

**Figure 5:**
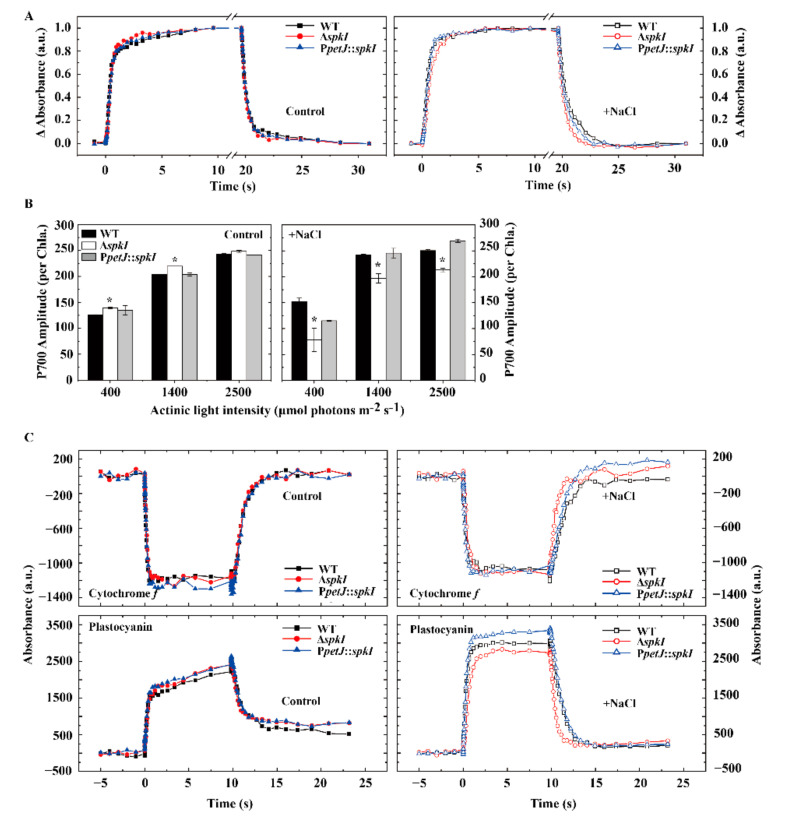
Oxidation and reduction of P700 and cytochrome *b*_6_*f* complex in WT, Δ*spkI* and P*petJ*::*spkI*. (**A**), Kinetics of P700 oxidation and reduction. All curves were normalized to their maximal signals; (**B**), Amount of active PSI reaction centers under different actinic light intensities; the error bar represents the standard deviation (S.D.) of 3 independent experiments. Asterisk (*) represents significant differences for Δ*spkI* vs. WT at the level of *p* < 0.05 via student’s *t* test. (**C**), Cytochrome *f* and plastocyanin oxidation and reduction curves.

As shown in Figure 5A, there was only minor difference in kinetics of P700 among three strains under control condition, whereas Δ*spkI* showed obviously slower P700 oxidation and faster P700^+^ re-reduction than those of WT and P*petJ*::*spkI* under high salt condition, as the time constants reflected in Table 3. The amplitude of absorbance change reflects the amount of active P700 reaction centers. Only marginal difference in the amounts of P700^+^ was observed among different strains under control condition. Under high salt condition, P700 amplitude of Δ*spkI* always showed a significantly lower level than those of WT and P*petJ*::*spkI* regardless of light intensities (Figure 5B), which was in line with PsaB content (Figure 2).

Investigation of cytochrome *b*_6_*f* complex activity of WT, Δ*spkI* and P*petJ*::*spkI* grown under both conditions showed similar changes to those in P700. In brief, a slower oxidation and a faster reduction of cytochrome *f* and plastocyanin were observed in Δ*spkI* cells grown under high salt condition, but difference under control condition was not significant (Figure 5C, Table 3).

### 3.5. The Redox State of PQ Pool in WT, ΔspkI and PpetJ::spkI

**Figure 6:**
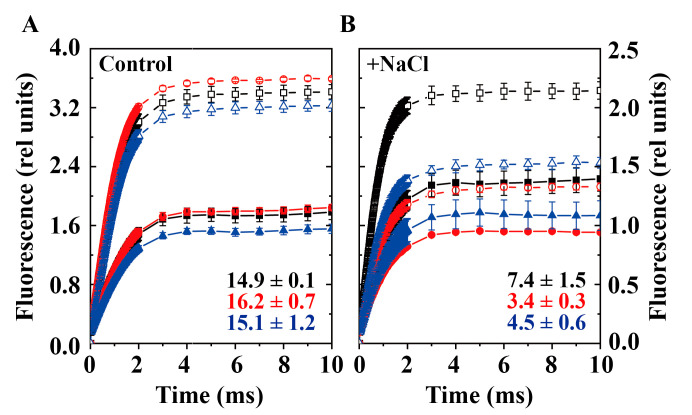
Estimation of redox state of PQ pool in WT, Δ*spkI* and P*petJ*::*spkI*. The redox state of PQ pool was measured in the absence (solid lines and symbols) or presence of DCMU (dashed lines and open symbols). The area between the curves (±DCMU) for each strain was shown in the plot as mean ± S.D. Cell suspensions were adjusted to 3 μg/mL Chl. *a* and were in darkness for 15 min before measuring. Cell suspensions were illuminated with 100 μmol photons m^−2^ s^−1^ actinic light for fluorescence induction. Black square symbol: WT, red circle symbol: Δ*spkI*, blue triangle symbol: P*petJ*::*spkI*. (**A**,**B**) represent the estimation of redox state of PQ pool of strains grown under control condition and high salt condition respectively. The curves are the average of at least three independent biological replicates with error bars.

Modifications of electron transfer in PSI and cytochrome *b*_6_*f* complex in Δ*spkI* (Figure 5) promoted us to investigate the redox state of PQ pool (Figure 6). The fluorescence induction curves showed that the percentage of area between curves (±DCMU) in Δ*spkI* was similar to that in WT and P*petJ*::*spkI* under control condition, but was much smaller in the cells grown under high salt condition. The situation of P*petJ*::*spkI* grown under high salt condition was between WT and Δ*spkI* suggested that the compensation of SpkI in P*petJ*::*spkI* was only partial in this experiment, which was tightly regulated by the transcription differences of gene *spkI* among three strains (Figure S2C). It was reported that the area between the curves (±DCMU) is at least partially proportional to the amount of dark-oxidized PQ [30,31]. Our data suggested a more reduced PQ pool in dark-adapted Δ*spkI* cells upon salt stress. This result was also supported by a much higher F_0_ level in Δ*spkI* under high salt condition (Figure 4B).

## 4. Discussion

### 4.1. SpkI Is Crucial for Optimal Photochemistry of PSII under High Salt Condition

Our results showed insignificance of SpkI under control condition concerning that the growth rate (Figure 1), oxygen evolution (Table 1), chlorophyll fluorescence traces (Figure 3) and major photosynthetic protein contents (Figure 2) were not inhibited in Δ*spkI*, which is consistent with previously reported results [32]. However, noticeably increased PSII content (D1, PsbI) and slightly higher PSII activity (Figure 2, Table 1) as well as slower Q_A_^−^ reoxidation (Table 2) suggested that the PSII centers in Δ*spkI* worked less efficiently than those in the WT under control condition. Although the phenomenon was not significant under control condition, inactivation of *spkI* led to a significant low Fv/Fm under high salt condition (Figure 1C), suggesting a more important role of SpkI upon salt stress. Moreover, reconstitution of gene *spkI* at a neutral site of the genome in the Δ*spkI* mutant restored the PSII maximal quantum yield (Figure 1C) and the growth (Figure 1D,E) under high salt condition further confirmed the phenotype by inactivation of gene *spkI*. Both oxygen evolution (Table 1) and Western blots (Figure 2) revealed a significant decrease in accumulation of PSII proteins in Δ*spkI* under high salt condition. The Q_A_^−^ re-oxidation (Figure 3) demonstrated certain modifications in electron transfer and photochemistry of PSII in Δ*spkI* under high salt condition. It was reported that under conditions of severe photoinhibition, the linear electron transport at PSII was strongly limited regardless of downstream acceptor availability [33]. Our data suggested that kinase SpkI could impair the electron transfer in PSII even grown under control condition, and cause a probable mild stress on PSII in Δ*spkI*. And slightly increased PSII content most probably was a compensation strategy for the vulnerable PSII to cope with environmental disturbances and maintain normal growth. When combined with additional NaCl, kinase SpkI could lead to more severe damage to PSII, and result in a strong decline of PSII amount and activity in Δ*spkI*. Accordingly, we propose that Ser/Thr kinase SpkI plays crucial roles in preserving optimal photochemistry of PSII under high salt condition.

### 4.2. SpkI Modifies Electron Transport of PSI and Cytochrome b_6_f Complex under High Salt Condition

Similarly, lack of SpkI affected the accumulation and activity of PSI and cytochrome *b*_6_*f* complex, especially under high salt condition (Figure 2). Slower oxidation and faster reduction kinetics of P700, cytochrome *f* and plastocyanin in Δ*spkI* grown under high salt condition (Figure 5, Table 3) indicated that the electron transfer of PSI and cytochrome *b*_6_*f* complex was modified. Such modification resulted in a faster cyclic electron transfer rate in the high-salt acclimated Δ*spkI* cells, which was consistent with higher amount of NdhK protein (Figure 2). Cyclic electron transport is physiologically essential for efficient photosynthesis [34]. Increased cyclic electron transport around PSI could provide energy for adaptation to high-salt conditions in cyanobacteria and eukaryotic algae [35]. Higher cyclic electron flow in Δ*spkI* further demonstrated that Δ*spkI* cells were more stressed than WT and may produce more ATP to facilitate cells to survive under stress conditions.

### 4.3. SpkI Is Involved in Regulating Redox State of PQ Pool under High Salt Condition

As the most essential electron carrier at the central position between two photosystems, the redox state of the PQ pool has been identified as an important parameter that can signal imbalances of photosynthesis by clamping the relative rates of electron release and uptake [36]. More reduced PQ pool in Δ*spkI* cells grown under high salt condition (Figure 6B) indicated a failure in balancing electron flow between PSII and its downstream (cytochrome *b*_6_*f* complex and PSI), which would cause damages to photosynthetic apparatus [37]. And the sharp decrease of protein amounts of PSII and PSI subunits in Δ*spkI* (Figure 2) could cause the electron flow imbalance and generate a more reduced PQ pool in Δ*spkI* under high salt condition. The reduction of PQ pool was caused by a preferential illumination of PSII by orange light principally absorbed by phycobilisomes [38]. More reduced PQ pool in Δ*spkI* mutant under high salt condition was most probably due to overexcitation of PSII. Enhanced cyclic electron flow (Figure 2) may also contribute to this phenomenon. We have noticed that the effect of SpkI on redox state of PQ pool was only partially complemented in P*petJ*::*spkI* under high salt condition, which was tightly associated with the transcriptional level of gene *spkI* in P*petJ*::*spkI* (Figure S2C).

### 4.4. SpkI Is Involved in Photoprotection Mechanisms in Synechocystis

Non-Photochemical Quenching (NPQ) and State 1-State 2 transitions are two important photoprotection mechanisms in cyanobacteria based on light harvesting antennae. The aim of NPQ is to decrease the energy arriving at the reaction center by thermal dissipation [39], which is tightly associated with a decrease in the rate of PSII photochemistry [40]. The trigger of NPQ in higher plants, mosses and green algae is related to a specific light-harvesting-chlorophyll antenna (LHCII) protein [41], while in cyanobacteria it is mediated by blue light [42] and orange carotenoid protein (OCP) [43]. An apparently higher NPQ was induced in Δ*spkI* cells grown under control condition (Figure 4A) further indicated that the PSII of the mutant requires more photoprotection efforts. The fluorescence recovery protein (FRP) was reported to regulate the function of OCP by their interactions [44]. More *ocp* transcripts accumulated in Δ*spkI* (Figure 4C) grown under control condition explained the higher quenching level (Figure 4A). Sharp declines of *apcA*, *apcB* and *cpcG1* transcripts in all strains after strong blue light could decrease the antenna size to reduce the light energy arriving at reaction centers. Higher levels of *apcA*, *apcB* and *cpcG1* transcripts accumulated in Δ*spkI* upon blue light illumination (Figure 4C) indicated that the mutant was over-excited. As the PSII in Δ*spkI* was mildly stressed under control condition, higher NPQ capacity would ensure the proper operation of the fragile PSII in the mutant. Given the fact that the PSII in Δ*spkI* was more susceptible to damage even grown under control condition, addition of extra high concentration of NaCl would boost the susceptibility and result in more severe photodamage to PSII. High salt treatment improved the NPQ level in general but with a smaller increment for Δ*spkI* (Figure 4A), which suggested that requirement of improving NPQ in Δ*spkI* has probably exceeded its limit and therefore the PSII functions could not be maintained anymore because of lacking of more efficient protections under high salt condition.

On the other hand, by preferential excitation of either reaction center, state transitions can balance energy distribution between photosystem I and photosystem II by monitoring the redox level of the PQ pool, and decrease the risks of the formation of dangerous reactive oxygen species [45]. In higher plants and green algae, state transition is related to LHCII and mediated by protein phosphorylation modifications [46,47]. In cyanobacteria, state transition occurs as the reversible binding of mobile phycobilisomes to PSII or PSI [45]. However, the involvement of phosphorylation in this process has been challenged by experiments performed on *Synechococcus elongatus* and *Synechocystis* in the presence of kinase and phosphatase inhibitors [28]. Participation of cytochrome *b*_6_*f* complex was also questioned in the same study, although, mutants defected in cytochrome *b*_6_*f* or PSI activity were deficient in state transitions [48,49]. Our results demonstrated that the state transitions could take place in all strains grown under both conditions (Figure 4B) implying the dispensability of SpkI as it was reported before [28]. However, we have noticed a limitation in the process of State 1 back to State 2 upon shift cells from blue light to red light in Δ*spkI* mutant. Plenty of studies revealed that state transition process in *Synechocystis* is related to gene *slr0328* [50], *cpcG1* [51], *pasK2* [52], gene *slr1128* and *isiA* [53,54]. The transcripts of *slr0328*, *cpcG1*, *psaK2*, *slr1128* and *isiA* varied not much among three strains in control, but were significantly down-regulated in Δ*spkI* upon shift to low blue light (Figure 4), indicating that the state transition in Δ*spkI* may not function efficiently as that in WT when the cells were in State 1, which consequently made a disturbance of light energy distribution between two photosystems in Δ*spkI* and eventually led to PSII overexcited. In addition, the crucial effect on regulation of state transitions by the redox state of PQ pool has been raised up [45]. A more reduced PQ pool in Δ*spkI* under high salt (Figure 6B) was correlated to State 2, which was characterized by lower photochemical activity of PSII, and high rate of P700 reduction as well as the low level of P700 oxidation, indicating high activity of cyclic electron transfer around PSI [55]. More reduced PQ pool was also probably due to inefficient redistribution of excitation energy to PSI by state transitions [56]. Thus, the performance of PSII, PSI and cytochrome *b*_6_*f* complex in Δ*spkI* under high salt condition implied that the equilibrium between the two photosystems was shifted.

As we illustrated above, the defects of Ser/Thr kinase SpkI would disturb the balance between photosystem II (PSII) and photosystem I (PSI). When cells grown under control condition, the disturbance only caused mild stress on PSII, and the normal growth of Δ*spkI* was sustained by a higher NPQ capacity and higher PSII activity. When cells grown under high salt condition, the excitation pressure got overwhelming on PSII, and led to a series of negative effects in Δ*spkI*. Although the higher respiration rate and CET in Δ*spkI* could compensate, it’s still not enough, and the growth of Δ*spkI* was impaired.

## 5. Conclusions

**Figure 7:**
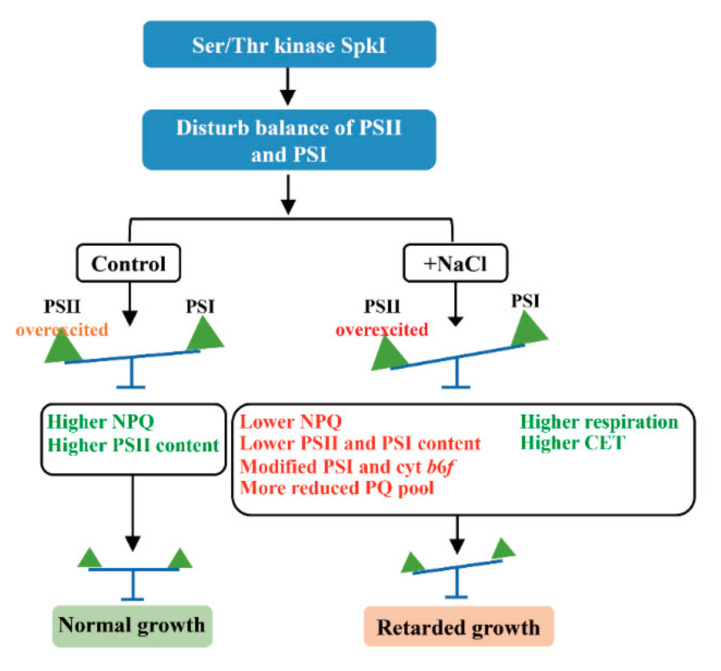
A brief working hypothesis of Ser/Thr kinase SpkI upon salt stress.

Combining all results above, we could evidence the role of Ser/Thr protein kinase SpkI in regulating photosynthetic efficiency in *Synechocystis* under high salt condition, although the specific functional mechanisms behind still need more efforts to be clarified. Despite all this, a bold hypothesis was proposed based on our results and a brief schematic was shown in Figure 7. When grown under control condition, disruption of Ser/Thr kinase SpkI caused mild stress on PSII, and Δ*spkI* was able to cope with the slight imbalance of excitation by increase of PSII and higher NPQ capacity to keep normal growth. When grown under high salt condition, additional salt stress exacerbated excitation pressure on PSII, and the imbalance of excitation distribution became more dangerous, which finally caused decreases of both PSII and PSI proteins as well as modifications of PSI and cyt *b*_6_*f* complex. Even though the enhanced cyclic electron flow around PSI and higher respiration rate could supplement energy when photosynthesis was declined in Δ*spkI*, it still led to retarded growth under high salt condition. In conclusion, Ser/Thr protein kinase SpkI is involved in maintaining high-effective photosynthesis in *Synechocystis* upon salt stress, thus it may play a role in high-salt adaptation mechanisms.

## Figures and Tables

**Table 1:** Photosynthetic and respiratory activities of WT, Δ*spkI* and P*petJ*::*spkI*.

Strain	Oxygen Evolution/Uptake (μmol O_2_·mg^−1^ Chlorophyll·h^−1^)			
	Net Photosynthesis	PSII Activity	PSI Activity	Respiration
Control				
WT	415 ± 1	734 ± 4	−185 ± 11	−32 ± 4
Δ*spkI*	410 ± 5	770 ± 10	−150 ± 6	−31 ± 1
P*petJ*::*spkI*	395 ± 10	713 ± 5	−165 ± 6	−32 ± 1
+NaCl				
WT	322 ± 9	759 ± 5	−158 ± 11	−48 ± 1
Δ*spkI*	259 ± 5	530 ± 17	−161 ± 4	−59 ± 2
P*petJ*::*spkI*	355 ± 4	729 ± 13	−168 ± 11	−51 ± 1

The activities of PSII and the net photosynthesis were measured as the steady-state oxygen evolution rate under saturating light. The PSI activity and respiration rate were measured as oxygen uptake under saturating light and in darkness after illumination for 1 min respectively. Values are given as a mean ± S.D. from three independent experiments.

**Table 2:** Kinetics of the flash-induced chlorophyll fluorescence of WT, Δ*spkI* and P*petJ*::*spkI* strains.

Strain	Fast Phase		Middle Phase		Slow Phase	
	A1 (%)	T1 (μs)	A2 (%)	T2 (ms)	A3 (%)	T3 (s)
Control						
WT	51 ± 0.4	681 ± 20	29 ± 1.6	4.7 ± 0.2	20 ± 2.0	9.1 ± 0.5
Δ*spkI*	54 ± 0.3	747 ± 1	27 ± 0.1	5.2 ± 0.1	19 ± 0.4	8.6 ± 0.1
P*petJ*::*spkI*	51 ± 1.1	633 ± 16	31 ± 1.0	3.9 ± 0.2	19 ± 0.4	7.1 ± 0.7
+NaCl						
WT	58 ± 2.4	530 ± 6	23 ± 1.6	6.5 ± 0.7	19 ± 0.8	5.5 ± 0.8
Δ*spkI*	47 ± 2.8	769 ± 39	32 ± 2.8	5.1 ± 0.3	20 ± 0.4	4.1 ± 0.2
P*petJ*::*spkI*	56 ± 1.6	580 ± 11	24 ± 0.4	5.6 ± 0.4	20 ± 1.3	4.2 ± 1.0

The results are shown as the means from three independent experiments ± S.D. A, Amplitude; T, time constant.

**Table 3:** Oxidation and reduction kinetics of cytochrome *f*, plastocyanin and P700 (shown as t_1/2_ (s)).

Growth Condition	Cytochrome *f*		Plastocyanin		P700	
	Oxidation	Reduction	Oxidation	Reduction	Oxidation	Reduction
Control						
WT	0.238 ± 0.005	1.351 ± 0.049	0.341 ± 0.002	1.175 ± 0.003	0.362 ± 0.002	0.585 ± 0.004
Δ*spkI*	0.335 ± 0.016	1.118 ± 0.047	0.438 ± 0.001	1.182 ± 0.006	0.590 ± 0.013	0.630 ± 0.010
P*petJ*::*spkI*	0.265 ± 0.001	1.386 ± 0.169	0.386 ± 0.001	1.110 ± 0.040	0.412 ± 0.005	0.552 ± 0.001
+NaCl						
WT	0.261 ± 0.002	1.712 ± 0.132	0.381 ± 0.007	1.533 ± 0.030	0.550 ± 0.004	0.885 ± 0.007
Δ*spkI*	0.738 ± 0.018	0.627 ± 0.004	0.651 ± 0.015	0.583 ± 0.005	0.918 ± 0.007	0.451 ± 0.010
P*petJ*::*spkI*	0.272 ± 0.001	1.416 ± 0.050	0.368 ± 0.002	1.290 ± 0.015	0.664 ± 0.001	0.774 ± 0.047

Results are given as means ± S.D.

## Data Availability

The data underlying this article are available in the article and in its online Supplementary Materials.

## Supplementary Materials

The following supporting information can be downloaded at: https://www.mdpi.com/article/10.3390/life12050713/s1, Figure S1: PCR testification of complete segregation of ΔspkI; Figure S2: Construction of the complementary strain and transcription analysis of gene spkI; Table S1: Primer pairs used in this study. Western blot uncropped images were also included as requested.
