# Supplementary material for: Ser/Thr Protein Kinase SpkI Affects Photosynthetic Efficiency in Synechocystis sp. PCC 6803 upon Salt Stress
Source: Life (Basel). 2022 May 10;12(5):713. doi: 10.3390/life12050713 (PMC9143257; doi:10.3390/life12050713)
Supplement: Supplementary file 1 [file life-12-00713-s001.zip › life-1697618-supplementary.pdf]

Supplementary Materials

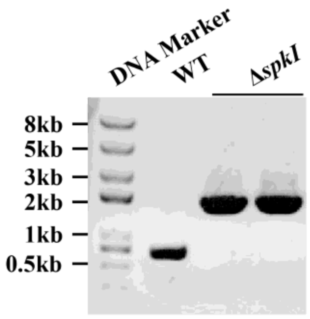

Figure S1 PCR testification of complete segregation of Δ*spkI*

The electrophoretic band in WT was 682bp, and the corresponding PCR product in Δ*spkI* should be 1655bp.

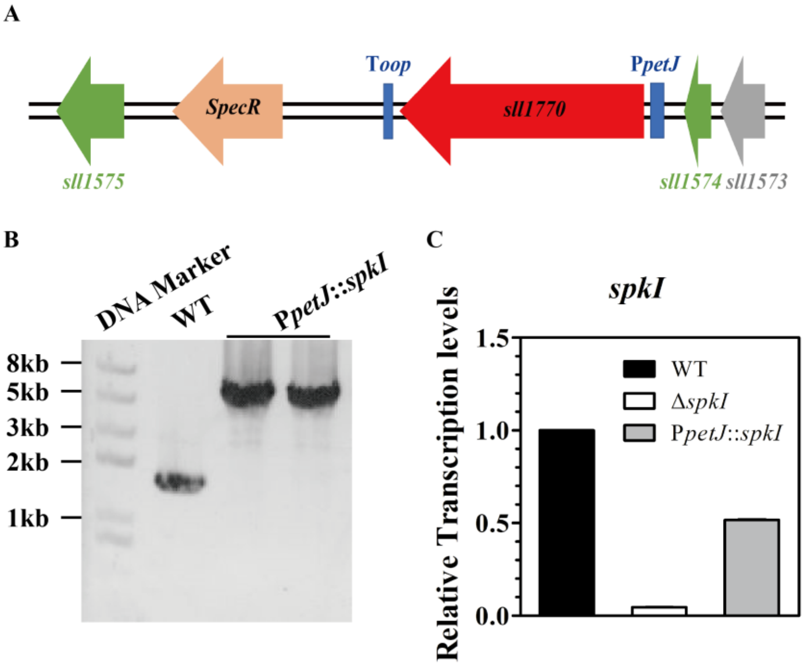

Figure S2 Construction of the complementary strain and transcription analysis of gene *spkI*

A, Cloning scheme of complementary strain P*petJ*::*spkI* ;

B, PCR verification of segregation of P*petJ*::*spkI*, the PCR product in WT and P*petJ*::*spkI* is 1512bp and 4600bp respectively ;

C, Relative transcription analysis of *spkI* gene in all strains.

Table S1 Primer pairs used in this study

Gene name	Forward primer (5'-3')	Reverse primer (5'-3')
ocp (*slr1963*)	ATTTTCCCTAACACCCTAGCG	TGATCTTCGGCATTGAGTTGG
frp (*slr1964*)	CAAAACGCCACGAAATTGATG	CCAGGGCCTTAATTTTAGATTGC
cpcG1 (*slr2051*)	AACTACCGCTTCGTTGAGCA	TAAAGGGCAATTCACCCGCT
apcA (*slr2067*)	AAATGTATCGTTCCCTCGGC	CGAAGTCAAAGTAGGCAGAGG
apcB (*slr1986*)	AAAGAAACCTACAACTCCCTCG	GCAGATGTAGTCGAGGTAAACG
slr0328	ACGCCCAGATTGACCAAG	CTTCCCTGCACCCGATAAC
psaK2 (*sll0629*)	CTTTGGCTTGCCGGAATTG	CTAGACCCAACACCATGCC
slr1128	TCCCCGTAACATTCTTTCCAC	CCGCCTCTAATCCTTCATCTAG
isiA (*sll0247*)	CAAACCTATGGCAACGACAC	TCAAACAGGGTAAAGGCTCC
rnpB (*slr1469*)	GGACTACCCAAAACACTGC	CAATAATCCCAGCTTGGCT
spkI-up	ACGCCGCCGCCGTTG	AGTGGTGGTCTGTTCCGTT
spkI-down	AAGGCACTATTACCCACCTG	CAAGCCAAATGGATTCGAGA
Δ*spkI* segregation	GGTCGCACCTATGTAATCCC	TTCACCACCACATCTTCGCC
spkI-ox	GTGTCTGCCCTATCCACTAA	TTAAAACATCCGGTCCTGT
P*petJ*::*spkI* segregation	AGGGGAAGGCTGGCTCTC	CAACCAGCGGGGACCATA

Western blot original images

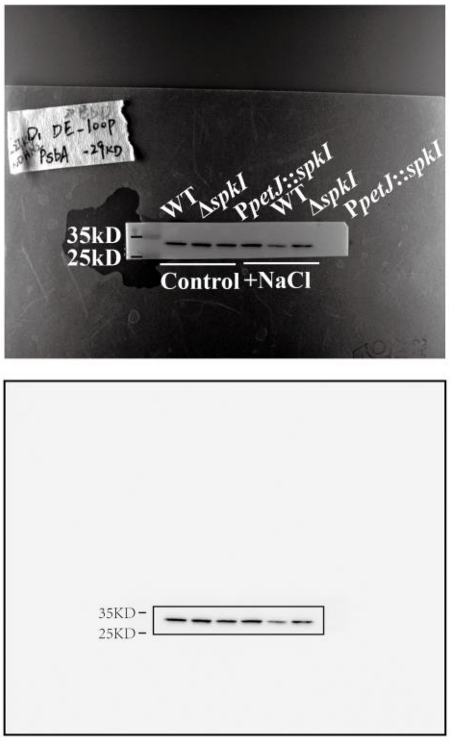

D1

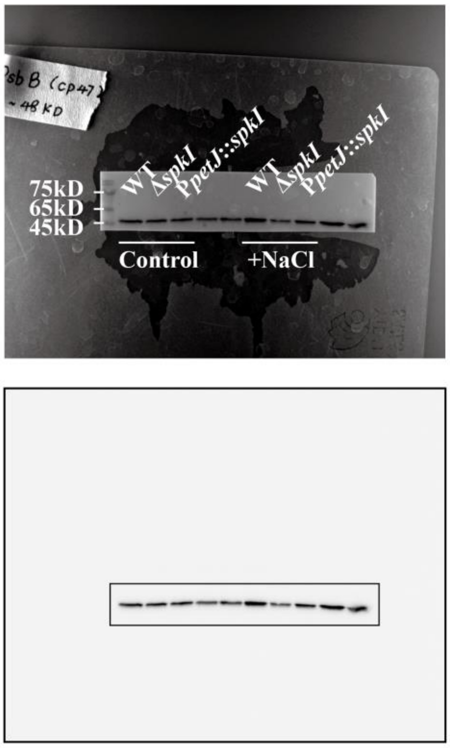
WT
ΔspkI
PpetJ::spkI
WT
ΔspkI
PpetJ::spkI
75kD
65kD
45kD
Control
+NaCl

PsbB

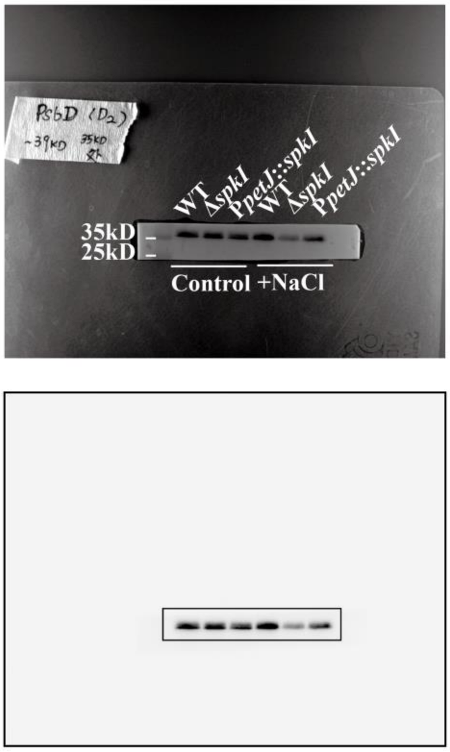
PsbD (D2)
~39KD 35KD
WT
Δspk1
PpetJ::spk1
WT
Δspk1
PpetJ::spk1
35kD
25kD
Control
+NaCl

PsbD

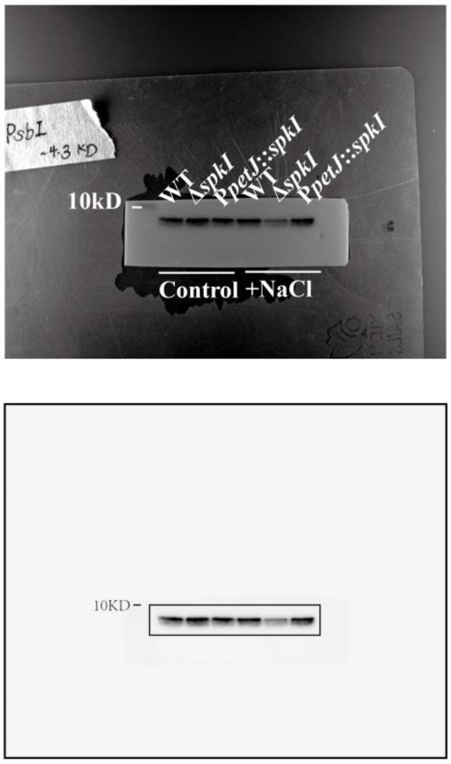
PsbI
~4.3 KD
10kD
WT
ΔspkI
PpetJ::spkI
WT
ΔspkI
PpetJ::spkI
Control
+NaCl
10KD

PsbI

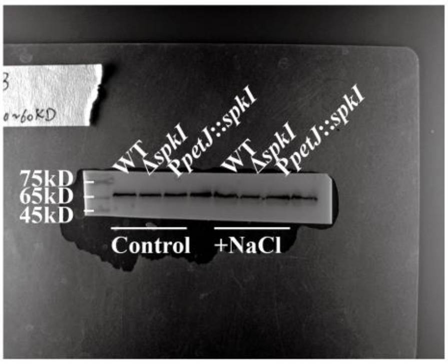
WT
ΔspkI
PpetJ::spkI
WT
ΔspkI
PpetJ::spkI
75kD
65kD
45kD
Control
+NaCl

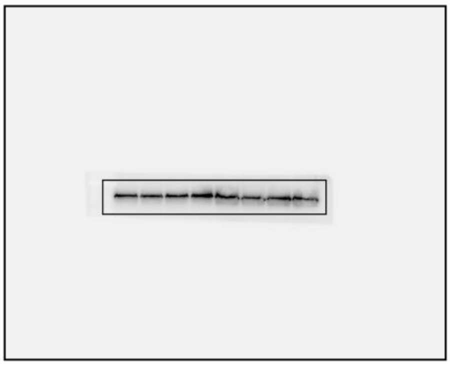

PsaB

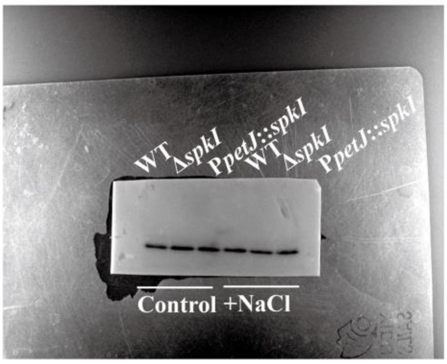

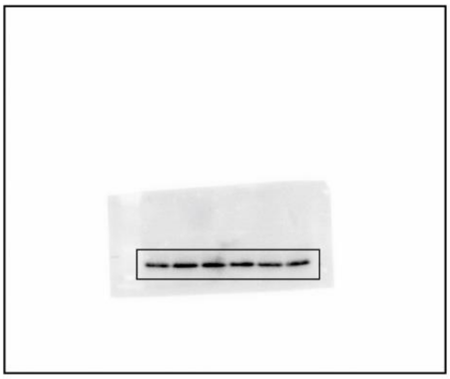

APC

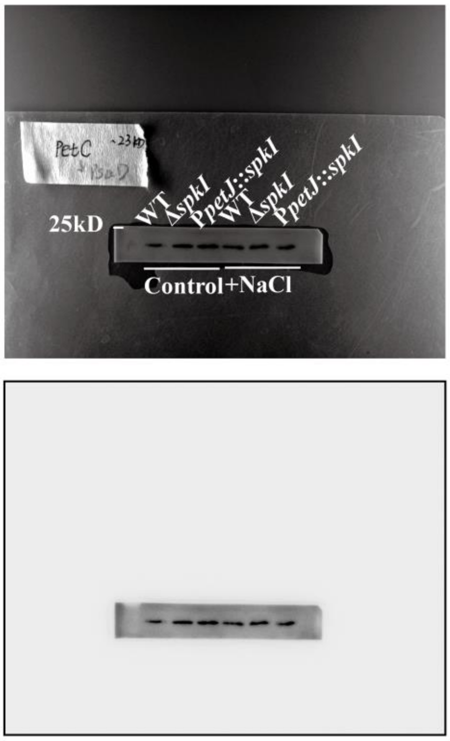
PetC
25kD
WT
ΔspkI
PpetJ::spkI
WT
ΔspkI
PpetJ::spkI
Control
+NaCl

PetC

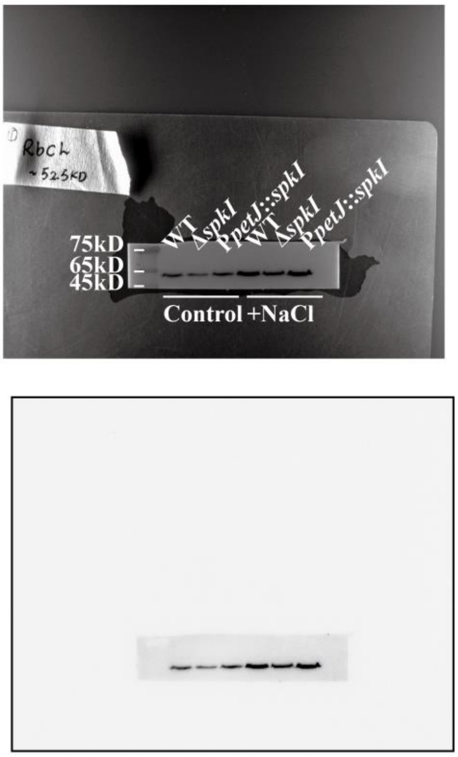
RbcL
~52.5KD
75kD
65kD
45kD
WT
ΔspkI
PpetJ::spkI
WT
ΔspkI
PpetJ::spkI
Control
+NaCl

RbcL

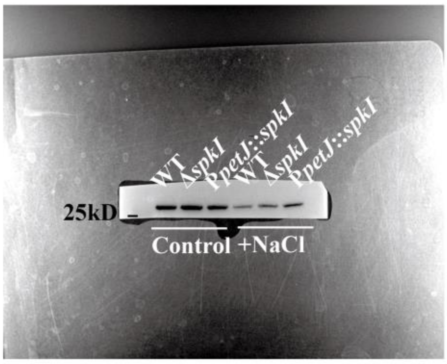

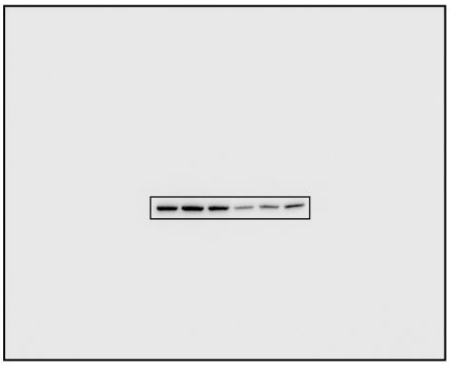

NdhK

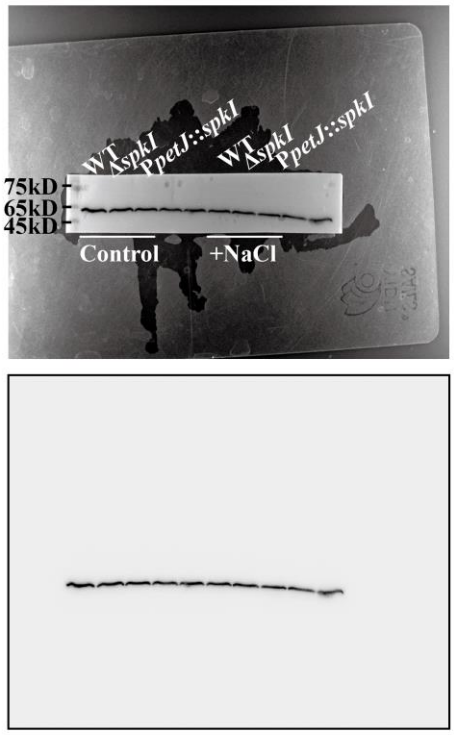
WT
ΔspkI
PpetJ::spkI
WT
ΔspkI
PpetJ::spkI
75kD
65kD
45kD
Control
+NaCl

AtpB
